# Metal-Free Radical Homopolymerization of Olefins and Their (Co)Polymerization with Polar Monomers

**DOI:** 10.3390/polym17212871

**Published:** 2025-10-28

**Authors:** Miguel Rosales-Guzmán, Enrique Saldívar-Guerra, Carlos Guerrero-Sánchez

**Affiliations:** 1 Centro de Investigación en Química Aplicada, Blvd. Enrique Reyna Hermosillo No. 140, Saltillo C.P. 25294, Coahuila, Mexico; 2 Universidad Tecnológica de Coahuila, Av. Industria Metalúrgica No. 2001, Ramos Arizpe C.P. 25900, Coahuila, Mexico; 3 Laboratory of Organic and Macromolecular Chemistry (IOMC), Friedrich Schiller University Jena, Humboldtstrasse 10, 07743 Jena, Germany; 4 Jena Center for Soft Matter (JCSM), Friedrich Schiller University Jena, Philosophenweg 7, 07743 Jena, Germany

**Keywords:** olefin, polar monomers, radical copolymerization

## Abstract

Currently, a significant percentage of the industrial production of polyolefins and olefin-based copolymers is performed via free-radical polymerization, requiring demanding reaction conditions (i.e., high pressure and temperature) or via catalytic coordination insertion polymerization relying on the use of transition-metal catalysts. In general, these catalysts are not compatible with polar monomers, are air-sensitive and have to be used in high concentrations, which may preclude their use in specific applications. On the other hand, metal-free radical polymerization is a more robust method in terms of compatibility with air and types of monomers, with the limitation of requiring demanding reaction conditions. Nevertheless, the pursuit for strategies to synthesize olefin-based polymers under milder reaction conditions via metal-free radical polymerization is an active area in polymer science. The revision of the state of the art of such approaches is the focus of this contribution.

## 1. Introduction

The current plastic market is mainly governed by the production of polyolefins such as polyethylene (PE) and polypropylene (PP) and copolymers derived thereof. Despite its low compatibility with polar materials, such polymers remain highly valuable due to their resistance to oxidation, durability and relatively low cost. The conventional industrial free-radical polymerization (FRP) of PE is carried out at high temperatures (>200 °C) and pressures (1000–3000 bar). These two reaction parameters can be used to “modulate”, to some extent, some properties such as molar mass and branching of the obtained materials [1]. In the case of PP and other olefins (octene, hexene, etc.), radical chain transfer to monomers mainly dominates over radical chain propagation, leading to very low polymer yields [2,3]. This is the reason why PP is mainly produced utilizing metal-based catalysts [4,5,6]. For example, an interesting strategy for the synthesis of PP and isobutylene (IB) homopolymers along with IB/Ethyl acrylate copolymers via Li+-catalyzed free-radical polymerization has been reported by Michl and collaborators [4,6]. These catalysts are, in general, difficult to remove from the final products or are incompatible with polar monomers to produce copolymers. Other systems use rare-earth semi-metal compounds, but they have been limited to the polymerization of ethylene [7]. Thus, the commercial production of olefin-based copolymers containing polar units derived from vinyl monomers, such as vinyl acetate (VA), is attained via FRP processes utilizing high temperatures (150–375 °C) and pressures (250–3000 bar). Control over the molar mass or topology of the obtained polymers becomes difficult under such reaction conditions. Hence, the development of less demanding or improved FRP processes for the homopolymerization of olefins and their copolymerization with polar vinyl monomers could be of economic interest for industry, as it could lead to the production of polymers of controlled molar mass, composition, and architecture. Diverse alternative solutions to these challenges have been investigated by different researchers, which is the focus of this review.

Therefore, this review highlights recent advances in the metal-free homopolymerization of olefins and their copolymerization with polar vinyl monomers, which utilize “mild” reaction conditions via FRP and/or reversible deactivation radical polymerization (RDRP) techniques such as nitroxide-mediated polymerization (NMP), reversible addition–fragmentation chain transfer polymerization (RAFT), iodine transfer polymerization (ITP) and enhanced spin capturing polymerization (ESCP). The main advantage of these polymerization techniques is their compatibility with polar and non-polar monomers, as well as their low sensitivity to oxygen. Although improvements in the metal-free atom transfer radical polymerization (ATRP) have been recently reported, they are limited to particular polar monomers, but still have not been reported for ethylene or other olefins [8]. Other methods of polymerization involving metals, such as the Ziegler–Natta method, are also beyond the scope of this review [9].

A review in the field of olefin polymerization has been recently published, highlighting mainly the polymerization of ethylene [10], whereas our contribution covers the polymerization of other systems involving different olefins and complements previous reports.

## 2. Homopolymerization of Ethylene

**Scheme 1:**
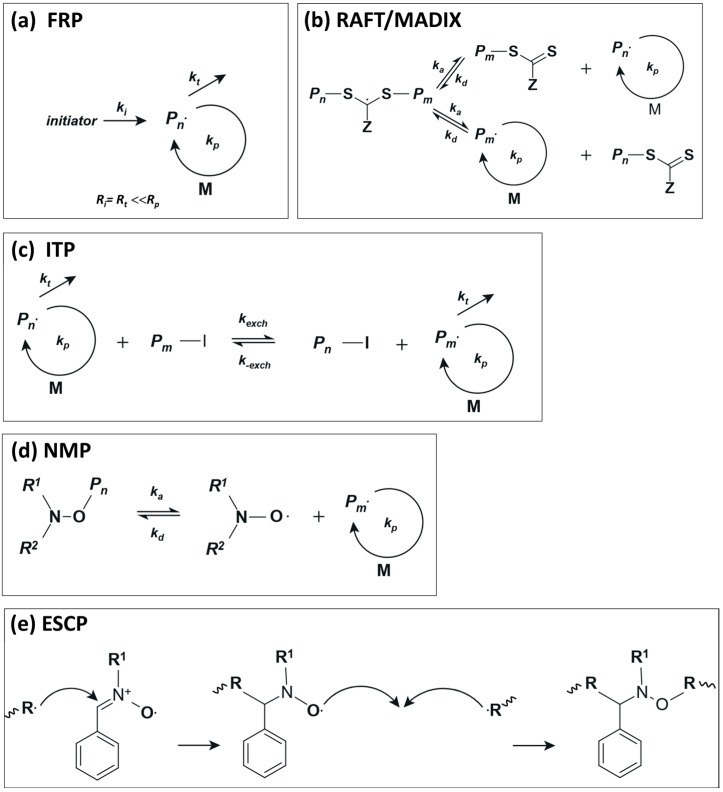
Simplified mechanisms of (**a**) FRP, (**b**) RAFT/MADIX, (**c**) ITP and (**d**) NMP polymerization. While in an FRP, termination and initiation events are very similar, in RAFT, ITP and NMP, the termination events are limited by a dynamic equilibrium between active and dormant species. (**e**) Shows the key mechanism in ESCP.

Scheme 1a shows a simplified mechanism in a classical free-radical polymerization. In the case of ethylene at moderate to low reaction conditions, the growing radical is not sufficiently stabilized, which results in an inefficient ethylene polymerization; the rate of propagation (R_p_) is small compared to the rate of termination (R_t_). However, as will be discussed below, the choice of solvent is a key factor for the homopolymerization of ethylene at moderate reaction conditions [11,12,13]. In the case of RAFT and MADIX (macromolecular design via the interchange of xanthates), both polymerization techniques follow the same mechanism and differ in the control agent, which is termed xanthate in the case of the MADIX technique [14]. The general mechanism in a RAFT/MADIX polymerization relies on a dynamic equilibrium between propagating radicals and dormant species according to the simplified mechanism shown in Scheme 1b. The concentration of dormant species is large compared to propagating radicals, which, essentially, limits the termination events and results in narrow molar mass distributions. In the case of ethylene, xanthates and dithiocarbamates are promising candidates to perform the ethylene polymerization under RAFT/MADIX conditions, although unwanted irreversible side fragmentation of the intermediate radical may preclude its broad application [15,16]. Iodine transfer polymerization is another strategy in which side reactions are much less favorable in comparison to the side reactions mentioned above for the RAFT polymerization of ethylene [17]. Scheme 1c shows the general mechanism of ITP, whose mechanism is based on degenerative chain transfer (DT). Meanwhile, NMP relies on the homolytic decomposition of an alkoxyamine, leading to a propagating radical ($P_{n}^{\cdot }$) and a controlling nitroxide R^1^R^2^NO^●^, according to Scheme 1d [18]. Finally, in ESCP, the control of the polymerization is achieved by the stabilization of the growing macroradicals via spin-trapping agents (usually nitrones), which are then transformed into a stable macronitroxide. The latter is then able to trap another growing radical to generate an alcoxyamine (mid-chain functionalized polymer); see Scheme 1d. This alcoxyamine can then behave as in conventional NMP under certain conditions, allowing further chain extension of the functionalized polymer [19].

**Figure 1:**
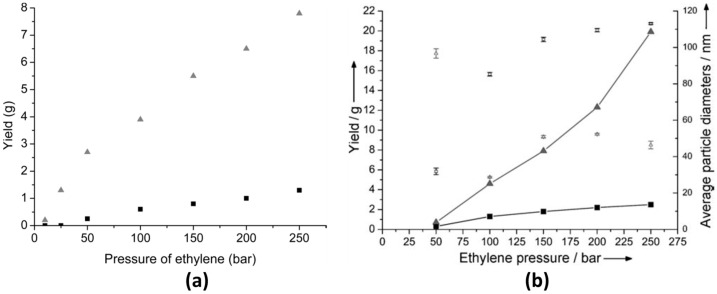
(**a**) Influence of pressure on the polymerization of ethylene at 70 °C in Tol (■) and tetrahydrofuran (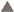). Reprinted with permission from Grau, E., Broyer, J.-P., Boisson, C., Spitz, R. & Monteil, V. “Free Ethylene Radical Polymerization under Mild Conditions: The Impact of the Solvent.” *Macromolecules* 42, 7279–7281 (2009). Copyright 2009 American Chemical Society [13]. (**b**) Free-radical polymerization of ethylene in aqueous dispersed medium: (■) yield and (□) average particle diameter versus ethylene pressure (80 mg AAPH, 50 mL water, 4 h at 70 °C under ethylene pressure); (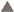) yield and (∆) average particle diameter versus ethylene pressure (80 mg AAPH, 50 mL water with 1 g L^−1^ CTAB, 4 h at 70 °C under ethylene pressure). Reprinted with permission from reference [20].

As mentioned above, ethylene can be successfully polymerized at moderate reaction conditions by the use of an appropriate solvent. For example, Monteil et al. [13] studied the polymerization of ethylene in the pressure range from 10 to 250 bar using toluene (Tol) and tetrahydrofuran (THF) as solvents; see Figure 1a. As shown in this figure, THF is a good candidate to achieve larger conversions (up to 40%) in comparison to Tol (up to 3%) at 70 °C and 250 bar using azobisisobutyronitrile (AIBN) as a radical initiator. However, due to radical chain transfer to solvent, the obtained molar masses were lower in THF (ca. 2400 g mol^−1^) compared to Tol (ca. 4300 g mol^−1^). This unusual influence of solvent was ascribed to the stabilization of the growing radical via interactions with the solvent [13]. Later, the scope of the study was broadened to include various organic solvents of different polarity and susceptibility to radical chain reactions [12]. From this study, it was reported that dimethylcarbonate (DMC), the polar solvent with the lowest radical chain transfer ability, yielded a material with a molar mass of 11,700 g mol^−1^. In contrast, a polymer with a molar mass value of 370 g mol^−1^ was obtained when butanone was used as a solvent, and this low molar mass value was attributed to the higher radical chain transfer ability of this solvent [12]. Furthermore, the emulsion polymerization of ethylene at 70 °C in the pressure range from 50 to 250 bar can lead to polymers with higher molar masses along with higher yields in comparison to the solvent polymerization described above [20]. In the presence of a cationic hydrosoluble initiator, the formed particles were stable, and the polymer yield increased as a function of pressure; see Figure 1b. The performance of the polymerization increased when cetyltrimethylammonium bromide was used as a surfactant and showed higher yields as compared to the reaction in the absence of surfactant (e.g., 40% solids content was reported at 250 bar). At those reaction conditions, the obtained PE displayed a melting temperature of ca. 100 °C, a crystallinity value of 31% and a molar mass of 1.19 × 10^5^ g mol^−1^ [20]. This work was later extended to the synthesis of negatively charged PE particles using ammonium persulfate (APS) as an anionic initiator and sodium dodecyl sulfate (SDS) as the stabilizing agent for the emulsion case. The pH value of the reaction, the stirring rate and pressure were key factors for the polymer yield. In particular, a stable emulsion was obtained at 150 bar using a stirring rate of 750 rpm, which led to a yield of 30% solids content and to a material of a high molar mass (i.e., 2.1 × 10^6^ g mol^−1^) [21].

Other contributions describe the aqueous dispersion of a Laponite nanocomposite with PE, where the influences of the initiator and clay nanoplatelets on the colloidal stability of PE particles and yield (up to 24%) were investigated [22,23].

Alternatively, the use of CO_2_ as a reaction medium has been considered for the homo and copolymerization of different olefins [24]. In the case of ethylene, the homopolymerization was investigated at temperatures and pressures below 100 °C and 300 bar, respectively, and testing three different radical initiators (i.e., AIBN, lauroyl peroxide (LPO), and benzoyl peroxide (BPO)). In the case of AIBN, it was observed that polymer yield and molar mass increased as a function of CO_2_ pressure [25].

**Figure 2:**
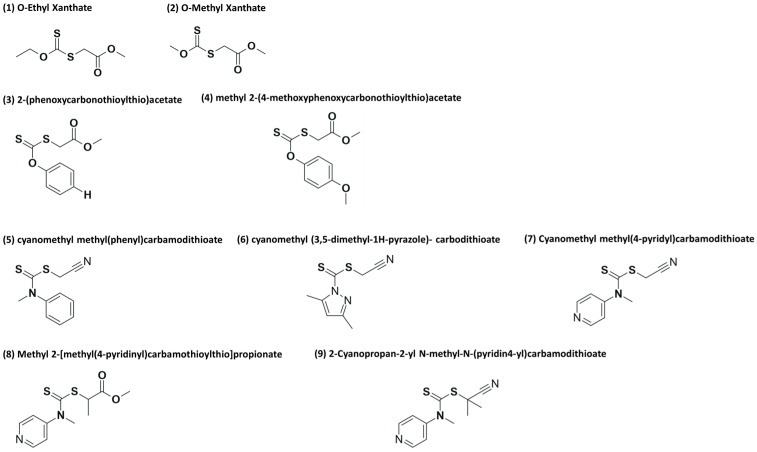
CTAs used for the RAFT homopolymerization of ethylene: alkyl xanthates (1) and (2), phenyl xanthates (3) and (4) and dithiocarbamates (5)–(9).

**Scheme 2:**
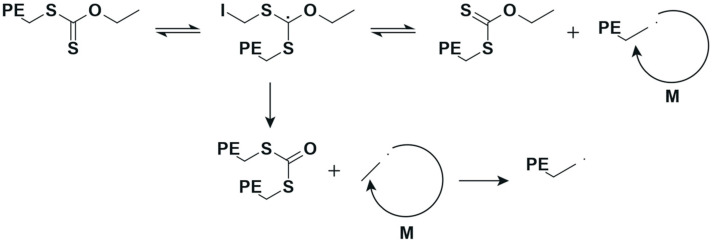
Overview of the reported side fragmentation in the RAFT polymerization of ethylene [16].

Otherwise, the controlled polymerization of ethylene via reversible addition–fragmentation chain transfer (RAFT) is challenging to say the least because only a limited amount of chain transfer agents (CTAs) are suitable, for example, xanthates and dithiocarbamates [15,16]. In the case of the RAFT polymerization of ethylene with two different CTAs, (1) *O*-ethyl xanthate and (2) *O*-methyl xanthate (see Figure 2 at 70 °C and 200 bar in DMC solution, interesting results were obtained regarding yield and dispersity. However, an unexpected side fragmentation of the intermediate radical (see Scheme 2) was noted according to nuclear magnetic resonance (NMR), resulting in different levels of end-group fidelity (“living” character of a polymerization). Chain extension reactions of PE with an 80% RAFT end-group fidelity allowed an increase in molar mass, but an increase in the *Đ* value from 1.7 to 3.4 was also attained.

Later, the assessment of aromatic xanthates and dithiocarbamates (CTAs 3–9 in Figure 2) was studied for the RAFT polymerization of ethylene at pressures and temperatures below 200 bar and 80 °C. Again, side fragmentation of the intermediate radical could not be avoided [15]. While dithiocarbamates yielded polymers with a molar mass of up to 3000 g mol^−1^ and *Đ* values in the range from 1.8 to 1.4, aromatic xanthates produced materials with a molar mass of up to 1000 g mol^−1^ and *Đ* values between 1.2 and 1.3. In addition, aromatic xanthates showed a more pronounced loss of chain ends than the cases of dithiocarbamates [15]. Furthermore, the ESCP of ethylene has also been reported, which led to mid-chain alkoxy-amine-functionalized PEs with a 90% functionality, where this high degree of functionalization allowed even the synthesis of block copolymers of styrene and ethylene [26]. Reasonable results in terms of polymer molar mass and yield were also obtained for the polymerization of ethylene in DMC at 70 °C and 200 bar. In another example, ITP, whose mechanism is based on degenerative chain transfer (DT), has also been proposed for the synthesis of PE [17]. Polymerization of PE via ITP seems to be more feasible in comparison to RAFT; however, high molar masses cannot be achieved. Table 1 summarizes important aspects of the contributions discussed in this section of the review.

## 3. Copolymerization of Olefins and Polar Vinyl Monomers

**Figure 3:**
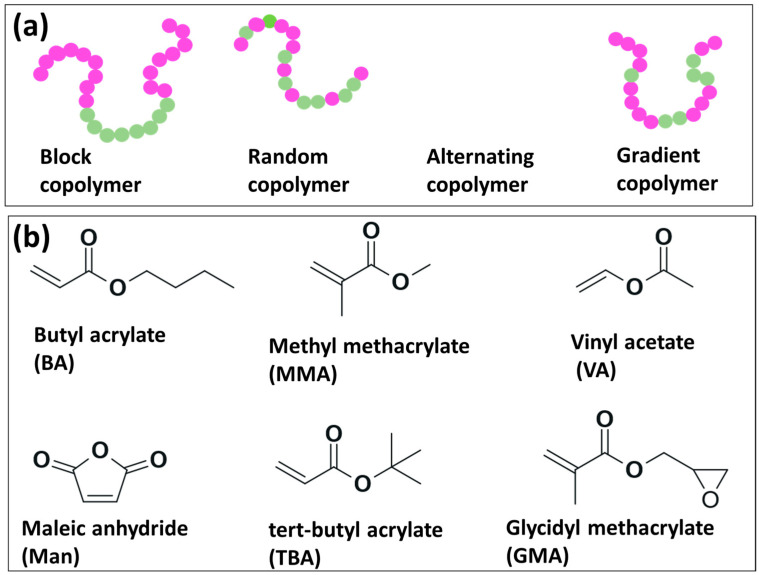
(**a**) Common types of linear copolymers and (**b**) chemical structure of most utilized polar monomers for the synthesis of olefin-based copolymers.

Copolymers of olefins bearing polar groups in their molecular structure are desirable for diverse applications that involve the interaction with other materials of polar nature or metals. These kinds of materials can be obtained, generally, by two approaches: (a) post-polymerization procedures or (b) direct copolymerization of the olefin with the corresponding polar monomers [27,28,29,30]. Linear copolymers can be categorized as block, random, alternating and gradient, as shown in Figure 3a. Although there are commercially available copolymers of olefins containing polar groups, such materials are typically synthesized utilizing demanding reaction conditions in terms of temperature and pressure [27]. Within this context, researchers have explored different strategies to use milder reaction conditions and, therefore, to favor the development of more robust polymerization methods. For instance, copolymerization reactions of olefins with polar comonomers have been investigated in dispersion [2,23,24], solution bulk [3,16,17,31,32,33,34,35,36,37,38,39,40,41,42,43,44], emulsion [45,46,47,48] and continuous methods [49]. In terms of the polymerization mechanism, polar monomers with 1-alkenes, ethylene, and propylene have been disclosed by different research groups employing different polymerization methods, such as FRP [32,48], (NMP) [32,36], RAFT [16,35,37,41] and ITP [35,38]. In Table 2, it can be observed the different polar/olefin copolymerization systems reported in the last two decades via metal-free radical polymerization mechanisms such as FRP, NMP, RAFT and ITP.

### 3.1. Copolymerization of 1-Alkenes with Polar Monomers

The homopolymerization of 1-alkenes is generally unsuccessful by metal-free-radical polymerization due to degradative chain transfer of allylic hydrogens; the allylic radical derived from the monomer has a low tendency to initiate a new polymer chain and eventually reacts with another allylic radical or a growing polymer chain, leading to a dead polymer [41]. Conversely, copolymers of methyl acrylate (MA) with 1-hexene, 1-octene and 1-decene were successfully polymerized by FRP, NMP and RAFT techniques [35,36]. In the case of RAFT polymerization, copolymers of MA with 1-hexene provided the better results in terms of olefin incorporation (20.2 mol%) and dispersity (1.07). In comparison, NMP copolymerizations resulted in olefin contents of 9.7 for 1-hexene (Đ = 1.1) and 8.0 mol% for 1-octene (Đ = 1.11). On the other hand, copolymerizations of Oct with BA and MMA led to similar incorporations of the olefin, 22 mol% for (Oct/BA) and 20 mol% for Oct/MMA, with slightly higher dispersities in comparison to the systems discussed above [41].

**Figure 4:**
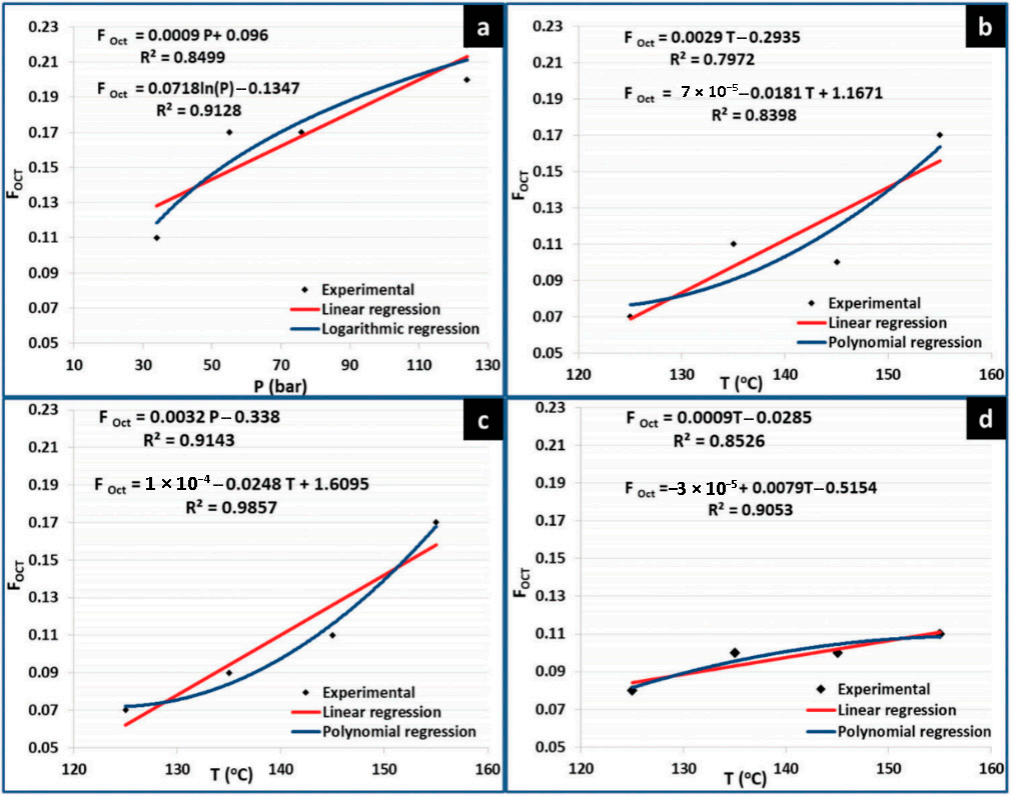
Copolymerization of GMA and Oct in CO_2_ as a reaction medium: influence of (**a**) pressure and (**b**–**d**) temperature on the olefin copolymer composition (F_Oct_). Reprinted with permission from Rosales-Guzmán M. [24].

While some researchers have used polar monomers such as butyl acrylate and methyl methacrylate [32,36,41,48], other works have focused on the utilization of more versatile polar monomers bearing functionalities that allow further modifications for modulating material properties, such as vinyl acetate [38], maleic anhydride (Man) [37], *tert*-butyl acrylate (TBA) [31] and glycidyl methacrylate (GMA) [3,24]; see Figure 3b. For example, FRP copolymerizations of 1-octene (Oct) with TBA were carried out to investigate the influence of type of initiator, comonomer feeding mode, comonomer feed ratio and reaction time on the copolymer composition, which led to the establishment of certain reaction conditions to obtain random copolymers with a maximum Oct composition of 37% [31]. The copolymerization of Oct with GMA was separately assessed in a mixture of organic solvents [3] and CO_2_ as a reaction medium [24]. In the latter report, the effect of temperature, feed comonomer composition and CO_2_ pressure was evaluated in relation to the content of Oct in the final copolymer. According to the report, olefin copolymer composition was influenced not only by temperature but also by CO_2_ pressure and by a combination of both parameters; a copolymer with a maximum Oct molar composition of 25% could be prepared, see Figure 4 [24]. The relatively low incorporation of the olefin was ascribed to chain transfer reactions to Oct monomer which is very common in the polymerization of α-olefins under FRP.

### 3.2. Copolymerization of Ethylene and Propylene with Polar Monomers

AIBN-initiated copolymerization of MA with ethylene (34.5 bar, 60 °C) and propylene (60 °C) led to random copolymers with 5.7 mol% ethylene (M_n_ = 2.8 × 10^5^, Đ = 9.0) and 21.5 mol% of propylene (M_n_ = 4.5 × 10^5^, Đ = 2.0). In comparison, the NMP copolymerization of MA with ethylene (48.3 bar, 120 °C) and propylene (16.9 bar, 120 °C) exhibited random copolymers with 13.6 mol% ethylene (M_n_ = 9 × 10^3^, Đ = 1.2) and 15.5 mol% propylene (M_n_ = 4.2 × 10^3^, Đ = 1.2). Although better control of the copolymerization was observed, considerably lower molar masses were reported [32].

Otherwise, the synthesis of copolymers containing ethylene-vinyl acetate (EVA) is probably one of the most investigated copolymerization reactions using different conditions of pressure and temperature and reaction mechanisms (e.g., FRP, RAFT and ITP) [11,16,34,39,40,44,45,50,51,52]. Concerning FRP, the copolymer composition of EVA polymers was explored as a response to varying temperature (70–85 °C), pressure (20, 30 bar), initiator concentration and stirring rate in emulsion copolymerizations. A copolymer with a maximum composition of ethylene of ca. 16% was synthesized at 30 bar and 75 °C [45]. The same copolymerization system was also studied at 70 °C in the pressure range from 50 to 125 bar in different organic solvents via FRP [39]. In this case, the most suitable solvents for the required application were THF and DMC, in which EVA copolymers could be synthesized with VA contents in the range from 4 to 32 mol% and molar masses from 1000 to 35,000 g mol^−1^. For the particular application of EVA as a cold flow improver, propanal was used as an additional chain transfer agent in combination with DMC for targeting specific molar masses [39].

**Scheme 3:**
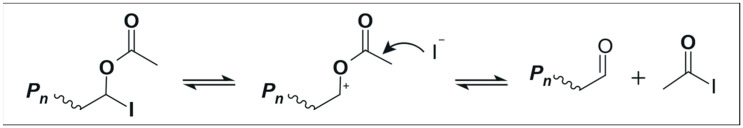
Schematics of chain-end degradation of EVA copolymers in the ITP technique when VA comprises the terminal unit. Image adapted from Figure S18 in the supporting information of reference [17].

Likewise, the controlled copolymerization of ethylene with VA using xanthates was similar to the PE homopolymerization described above (see Scheme 2) in which side fragmentation of the intermediate radical could not be avoided [16,33]. However, the use of dithiocarbamates can not only reduce this side fragmentation, but it can also allow the synthesis of three-arm EVA star copolymers [33]. In the same line, a poly(ethylene oxide) (PEO) homopolymer functionalized with dithiocarbamate ends was used for the successful synthesis of PEO-*b*-PE block copolymers [51]. On the other hand, the ITP technique has allowed the synthesis of EVA-*b*-PE and EVA-*b*-EVA copolymers [17], along with four-arm EVA star copolymers [43]. Although the ITP technique does not lead to side fragmentation of the intermediate radical as in the case of RAFT polymerization, it can lead to chain-end degradation when VA comprises the terminal unit; see Scheme 3.

## 4. Conclusions and Perspectives

Different strategies for the synthesis of homo and copolymers based on olefins and polar vinyl comonomers have been reported over the last two decades, utilizing different metal-free FRP and RDRP methods. This has allowed the synthesis of copolymers of 1-alkenes with polar monomers, such as MA, MMA, GMA, BA and TBA, among others, with different olefin copolymer composition using relatively mild reaction conditions. Furthermore, methods for the synthesis of homo and copolymers of ethylene with VA and BMA using milder reaction conditions have also been developed. Despite these advances, such strategies are still not utilized at industrial scales due to the low polymer yields and molar masses obtained and/or to the undesired odors/colors associated with the use of specific RAFT agents. However, FRP, RAFT and ITP techniques are still attractive alternatives for continuing to develop improved copolymerization systems of olefins with polar monomers due to their compatibility with a broad range of monomers, solvents and reaction conditions.

## Figures and Tables

**Table 1:** Summary of the homopolymerization of ethylene under different reaction conditions.

Entry/Ref	Initiator	Method ^†^	Solvent	Yield (g)/%Conv	P (bar)	T (°C)	M_n_^a^ (g/mol)	Đ ^b^
1/[13]	AIBN	FRP	Tol	1.3	250	118.7	4320	1.75
2/[13]	AIBN	FRP	THF	7.8	250	115.6	2410	1.99
3/[12]	AIBN	FRP	Tol/THF/DEC	4.1	100	70	5970	2
4/[25]	BPO	FRP	CO_2_	1.2	80	45	5000	1.6
5/[16]	AIBN	RAFT	DMC	5.76	200	70	1980	1.9
6/[15]	AIBN	RAFT	DMC	≅2	200	70	≅2400	≅1.6
7/[26]	AIBN	ESCP	DMC	5.3	200	70	11,500	4.3
8/[17]	AIBN	ITP	DMC	2.3	80	70	8000	1.7–3.5
9/[20]	AAPH	FRP	dispersion	19.9	250	70	119,000	6.3
10/[21]	AAPH	FRP	emulsion	15.17	200	80	2.1 × 106	2.4

^†^ Free radical polymerization (FRP), reversible addition–fragmentation chain transfer (RAFT), iodine transfer polymerization (ITP), enhanced spin capturing polymerization (ESCP). ^a^ Experimental molar mass. ^b^ Molar mass dispersity.

**Table 2:** Summary of the different copolymerization systems of olefins and polar monomers.

Entry/Reference	System ^†^	Method ^‡^	Year
1/[32]	MA with	FRP, NMP, RAFT, ITP	2004
	1-hexene		
	Oct		
	Ethylene		
	Propylene		
2/[41]	MMA/Oct	RAFT	2004
	MMA/BA		
4/[36]	MA with	NMP	2004
	1-hexene		
	Oct		
	Ethylene		
	Propylene		
5/[38]	Vinyl acetate with	ITP	2005
	Oct		
	Oct		
	1-decene		
	ethylene		
6/[37]	Man/1-decene	RAFT	2008
7/[31]	TBA/1-octene	FRP	2010
8/[34]	Ethylene with	RAFT/MADIX	2017
	HEVE		
	NVCL		
	VTFAc		
9/[3,24]	GMA/Oct	FRP	2018, 2022
10/[42]	BMA/Oct	RAFT	2019
11/[46,47]	Ethylene/BMA	RAFT, FRP	2019
12/[11,16,39,40,45,50,51,52]	Ethylene/VA	RAFT, MADIX, FRP, ITP	2020, 2021, 2023, 2013, 2023, 2024

^†^ Methyl methacrylate (MMA), 1-Octene (Oct), Butyl acrylate (BA), Maleic Anhydride (Man), Tert-butyl acrylate (TBA), 2-hydroxylethyl vinyl ether (HEVE), N-vinylcaprolactam (NVCL), vinyl trifluoroacetate (VTFAc), Glycidyl methacrylate (GMA), Butyl methacrylate (BMA). ^‡^ Free radical polymerization (FRP), Nitroxide-mediated polymerization (NMP), Reversible addition–fragmentation chain transfer (RAFT), Iodine transfer polymerization (ITP), Macromolecular design via the interchange of xanthates (MADIX).

## Abbreviations

The following abbreviations are used in this manuscript:

AAPH	2,2-azobis(2-amidinopropane)dihydrochloride
AIBN	Azobisisobutyronitrile
APS	Ammonium persulfate
ATRP	Atom transfer radical polymerization
BA	Butyl Acrylate
BMA	Butyl methacrylate
BPO	Benzoyl peroxide
CTAs	Chain transfer agents
Đ	Dispersity
DEC	Diethylcarbonate
DMC	Dimethylcarbonate
DT	Degenerative chain transfer
ESCP	Enhanced spin capturing polymerization
EVA	Ethylene-vinyl acetate
FRP	Free-radical polymerization
GMA	Glycidyl methacrylate
HEVE	2-hydroxylethyl vinyl
IB	Isobutylene
ITP	Iodine transfer polymerization
LPO	Lauroyl peroxide
MA	Methyl acrylate
MADIX	Macromolecular design via the interchange of xanthates
Man	Maleic anhydride
MMA	Methyl methacrylate
Mn	Molar mass
NMP	Nitroxide-mediated polymerization
NMR	Nuclear magnetic resonance
NVCL	N-Vinylcaprolactam
Oct	1-octene
PE	Polyethylene
PP	Polypropylene
RAFT	Reversible addition–fragmentation chain transfer
RDRP	Reversible deactivation radical polymerization
SDS	Sodium dodecyl sulfate
TBA	Tert-butyl acrylate
THF	Tetrahydrofuran
Tol	Toluene
VA	Vinyl acetate
VTFAc	Vinyl trifluoroacetate.
